# The technological proposal based on enhanced recovery after cardiac surgery for changing traditional care in Latin America: REPLICCAR III Study Protocol

**DOI:** 10.1371/journal.pone.0338301

**Published:** 2025-12-31

**Authors:** Gabrielle Barbosa Borgomoni, Pedro Horigoshi Reis, Guilherme Pinheiro, Fernando Faglioni Ribas, Gilmara Silveira da Silva, Valquíria Pelisser Campagnucci, Mário Issa, Pedro Silvio Farsky, Melina Moroz Barg, Marcos Gradim Tiveron, Luiz Fernando dos Reis Falcão, Roger Daglius Dias, Luiz Augusto Ferreira Lisboa, Fabio Biscegli Jatene, Omar Asdrúbal Vilca Mejia

**Affiliations:** 1 Departamento de CardioPneumologia, Disciplina de Cirurgia Cardiovascular, Unidade Cirúrgica de Qualidade e Segurança, Instituto do Coracao, Hospital das Clinicas HCFMUSP, Faculdade de Medicina, Universidade de Sao Paulo, Brazil; 2 Departamento de Cirurgia Cardiovascular, Beneficiencia Portuguesa de Sao Paulo, Brazil; 3 Departamento de Cirurgia Cardiovascular, Irmandade da Santa Casa de Misericórdia de Sao Paulo, Sao Paulo, Brazil; 4 Divisão de Cirurgia, Instituto Dante Pazzanese de Cardiologia, Sao Paulo, Brazil; 5 Departamento de Cirurgia Torácica e Cardiovascular, Irmandade da Santa Casa de Misericórdia de Marília, Marilia, São Paulo, Brazil; 6 Departamento Anestesiologia, Dor e Medicina Intensiva, Disciplina de Anestesiologia e Dor, Escola Paulista de Medicina EPM – UNIFESP, Sao Paulo, Brazil; 7 Department of Emergency Medicine, Harvard Medical School, Boston, Massachusetts, United States of America; Federal University of Santa Maria: Universidade Federal de Santa Maria, BRAZIL

## Abstract

The length of hospital stay following cardiac surgery remains a significant challenge. Enhanced recovery protocols, alongside the use of health applications, offer promising potential for safely and effectively reducing postoperative length of stay. The *Registro Paulista de Cirurgia Cardiovascular* III (REPLICCAR III) study, registered at Clinicaltrials.gov (NCT06786819), aimed to investigate the impact of an optimization application integrated within an optimized recovery-based protocol on postoperative hospitalization duration in patients undergoing Coronary Artery Bypass Graft Surgery (CABG) in the state of São Paulo.

## Introduction

Advances in cardiac surgery, driven by large databases, risk scoring systems, and technical innovations, have been significant [1–3]. However, the length of postoperative hospital stay remains a persistent challenge, especially within the Brazilian Unified Health System, where the average stay for coronary artery bypass graft (CABG) surgeries is approximately 11.8 days [4]. This issue is exacerbated by the lack of patient-centered protocols and misalignment within care teams [5–7].

Protocols based on the Enhanced Recovery After Cardiac Surgery (ERACS) concept aim to optimize care processes through a multidisciplinary, evidence-based approach, seeking to reduce both metabolic and psychological stress while fostering patient engagement in the recovery process [1,5,8]. Although promising, its implementation faces challenges related to management and adherence [9,10].

Digital platforms have shown potential to optimize outcomes by enhancing communication among healthcare teams and allowing better monitoring of care processes, thereby supporting adherence to protocol metrics [11–14]. These platforms have also demonstrated positive impacts on the patient and family education [15–17]. Within the context of rapid recovery, Schlund et al. [18] published a study on the use of a mobile application for patients undergoing elective colorectal surgery under the Enhanced Recovery After Surgery (ERAS) program, observing greater adherence to medication and shorter hospital stay, consistent with findings from other studies [19,20]. With the increasing use of mobile devices, health applications emerge as a promising means of promoting and facilitating health education.

In this context, the *Registro Paulista de Cirurgia Cardiovascular* III (REPLICCAR III) study seeks to analyze the impact of integrating a process customized application within enhanced recovery care pathways based on the concept of optimized recovery on postoperative hospitalization for patients undergoing CABG in the state of São Paulo, through the stepped-wedge cluster randomized design.

## Methods

**Fig 1:**
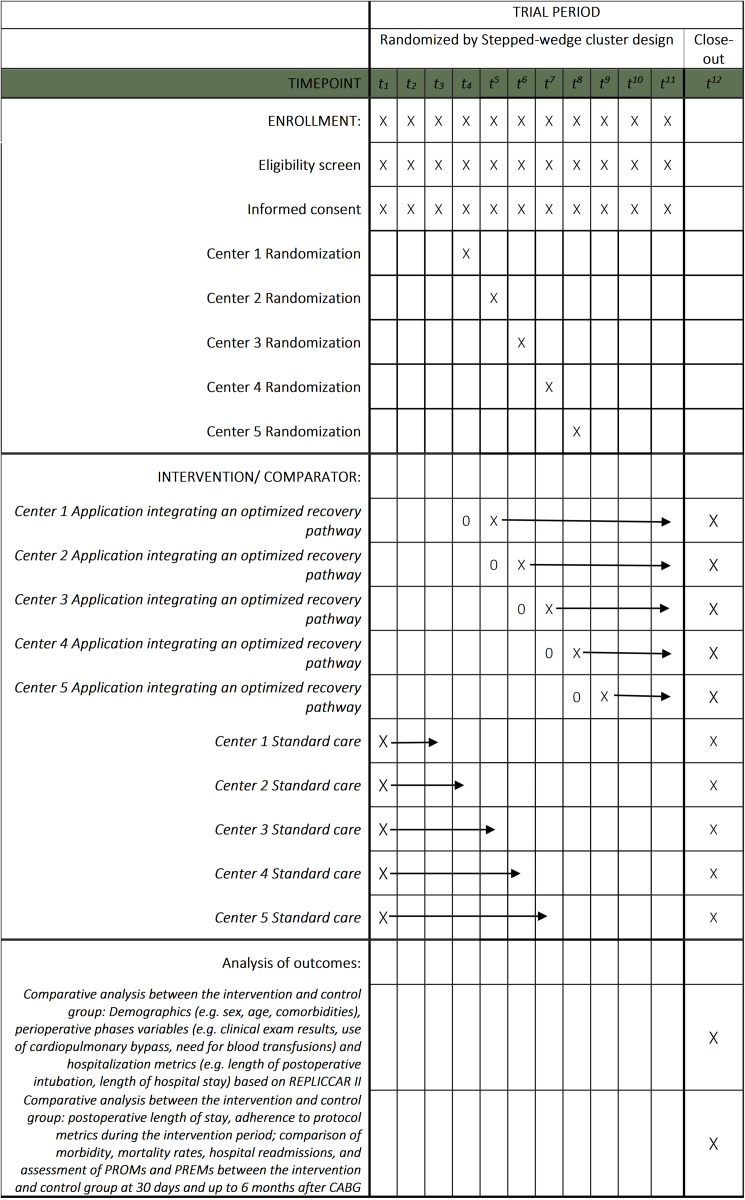
Schedule of enrollment, interventions, and assessments of REPLICCAR III project activities. Based on SPIRIT guidelines and recommendations [21,22]. Circle indicates training and implementation period, Arrow indicates continuous delivery of intervention. REPLICCAR III: Registro Paulista de Cirurgia Cardiovascular III; PREM: Patient-reported experience measures; PROM: Patient-reported outcome measures; CABG: Coronary artery bypass grafting.

A prospective, multicenter, randomized study using the stepped-wedge cluster design to analyze the impact of an application integrating an optimized recovery pathway, compared with standard care, on postoperative length of stay in patients undergoing CABG across five reference hospitals in the state of São Paulo. Patient recruitment and data collection began on 10 March 2025 (version 1, N = 252) and are expected to continue for one year to reach the target sample size. Therefore, the study is currently in the recruitment and data collection phase (Fig 1). It is registered at Clinicaltrials.gov (NCT06786819).

Participation in the study required approval from the executive board of each hospital to ensure that multiprofessional teams strictly adhere to the established protocols in the intervention group. A project representative at each participating institution will be responsible for obtaining informed consent, overseeing the patient’s perioperative course, and issuing quarterly progress reports to the coordinating center. The coordinating institution will monitor protocol adherence (in real time using the app) and ensure accurate data collection at each participating center monthly and through quarterly audits.

**Fig 2:**
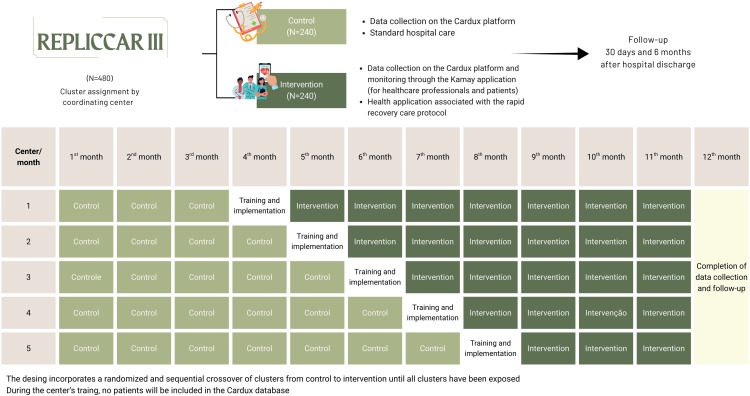
Study flowchart and timeline of REPLICCAR III project activities. REPLICCAR III: Registro Paulista de Cirurgia Cardiovascular III.

Fig 2 shows the study flowchart and timeline of project activities.

The Standard Protocol Items: Recommendations for Interventional Trials (SPIRIT 2025 Checklist) [21,22] is provided in figure in S1 File.

### Randomization design

Randomization of hospitals/reference centers (clusters) will be performed at the beginning of the project. Allocation will be carried out by an independent statistician and kept confidential by the study coordinating center. Hospitals will be informed of their assignment to the intervention group one month before the training of their multidisciplinary teams on the application incorporating the study protocols.

Each cluster will be randomly assigned to the intervention group in a staggered fashion:

- **Control period:** While the hospital is not randomized to the intervention group, data will be collected from the “control” period, consisting of observational data from standard care practices.
- **Implementation and training period:** Data from this period will not be analyzed, as it serves as an adaptation phase. Training on the application and optimized recovery protocols will be carried out through classes, meetings, and training sessions (with support from the STRATUS Center for Medical Simulation, Brigham Health) to ensure correct system use and adherence to the care protocol, using the intervention of *Tempos Certos* Protocol [23], based on Enhanced Recovery After Cardiac Surgery (ERACS) [24] concepts adapted to the context of the Brazilian Unified Health System.
- **Intervention period:** After the implementation period, collected data will be assigned to each hospital’s “intervention” group. As the project progresses, periodic meetings will be held to address challenges and provide updates on care at each institution.

### Follow-up

All patients included in the analysis will be followed up at 30 days and 6 months post-discharge, via telephone call, to assess hospital readmission rates, morbidity, patient-reported experience measures (PREM) [25,26], patient-reported outcome measures (PROM) adapted from the model by Ben-Ali et al. [27], as outlined in fil Table in S2 Table, and mortality.

Variables from the PREM and PROM instruments are collected in electronic forms dedicated to the REPLICCAR III project in REDCap, via telephone interviews conducted by the study’s technical research trainee and trained undergraduate research students [28]. All personnel were trained and instructed by the coordinating center to perform these activities, under the supervision of each responsible investigator. To mitigate loss to follow-up, several strategies were implemented, including providing patients with informational leaflets alongside the informed consent form and before hospital discharge, with follow-up call schedules and the names of study personnel. Information is reinforced to patients and their relatives during hospitalization.

An independent adverse event committee will follow up patients to track and report any adverse events and evaluate their potential relationship to the study design.

### Data storage and intervention application

All data will be securely stored online on dedicated, user-friendly platforms developed for the REPLICCAR III project in Cardux, REDCap, and Kamay. Both data storage and intervention platforms comply with the Brazilian General Data Protection Law (LGPD – Law no. 13.709/2018) [29], which regulates the processing of personal data in Brazil, protecting the fundamental rights to freedom and privacy for study participants and healthcare professionals using the platforms.

A confidentiality agreement will govern data use, ensuring alignment with the study objectives. The study will be conducted and monitored by an independent committee formed for data management and storage. All authors are required to sign a commitment statement affirming the accuracy of the collected data and adherence to the study protocol, although reviews and comments may be submitted throughout the study.

### Database

**Fig 3:**
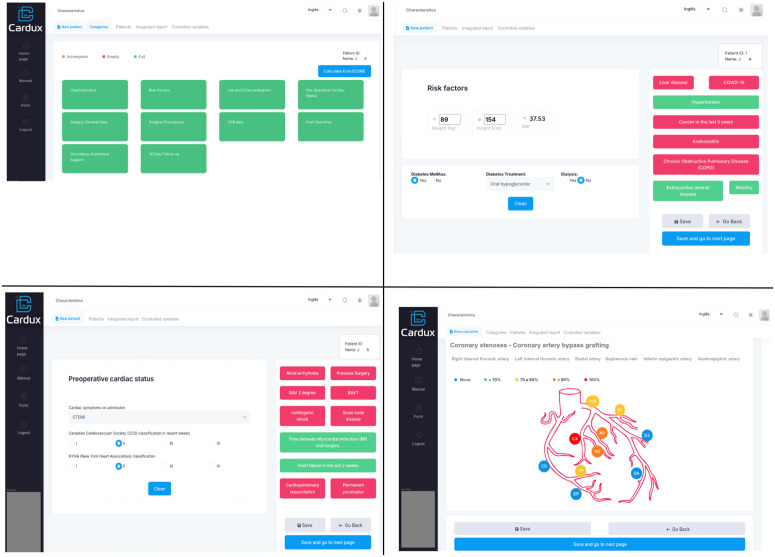
Cardux database REPLICCAR III dashboard.

The Cardux platform (https://cardux.net/, Fig 3) and REDCap [30] will be used for secure data storage, accessible by username and password for both study phases. Variables collected during the study will be based on insights from the REPLICCAR II project [31], addressing data related to patient demographics (e.g., gender, age, comorbidities), pre-, intra- and postoperative phases (e.g., clinical exam results, use of cardiopulmonary bypass, need for blood transfusion, complications), as well as hospitalization metrics (e.g., length of surgical procedure, postoperative intubation time, length of hospital stay). In addition, follow-up data will be collected at 30 days and 6 months post-discharge, including variables such as need for readmission, further interventions, incidence of acute myocardial infarction, mortality, and variables related to PREM and PROM analysis.

### Intervention application

Prior to the intervention phase, participating hospitals will complete a one-month training period to familiarize their staff with the platform and the *Tempos Certos* pathway, based on Enhanced Recovery After Cardiac Surgery [23]. The Kamay application (https://www.projetokamay.com.br/) will be used only during the intervention period, associated by Cardux database, with access protected by usernames and passwords, for real-time process monitoring.

**Fig 4:**
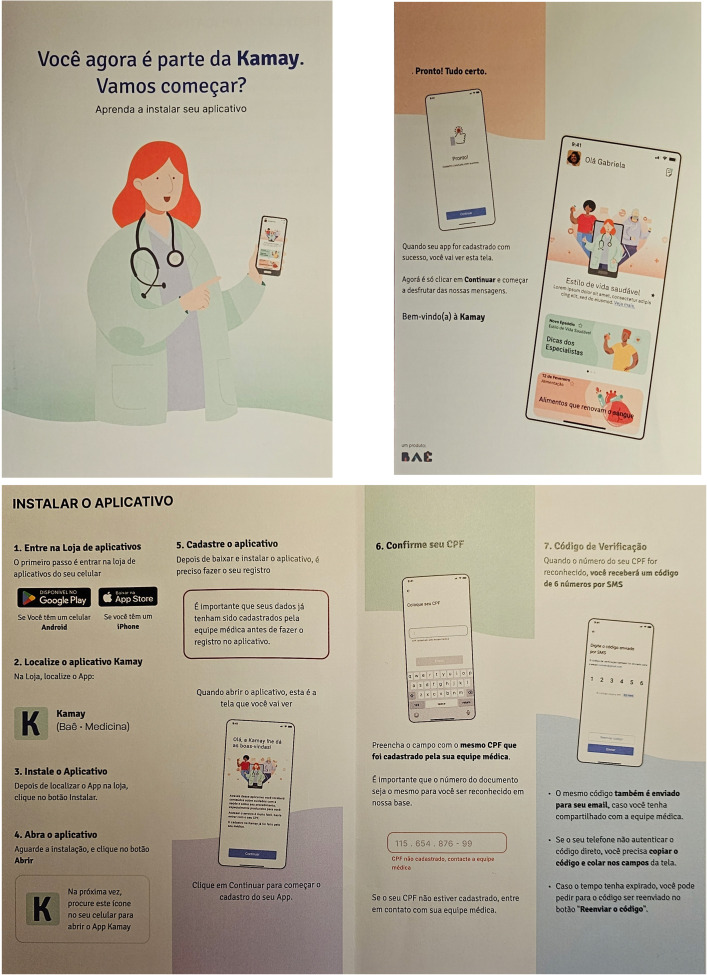
Pamphlet provided to intervention patients.

Patients will be introduced to the Kamay application and assisted with downloading and accessing it by the REPLICCAR study group at each site during recruitment. Additionally, every patient will receive an informational pamphlet (Fig 4) containing step-by-step instructions for downloading and using the application.

The application comprises three modules:

- Education of patients and families

**Fig 5:**
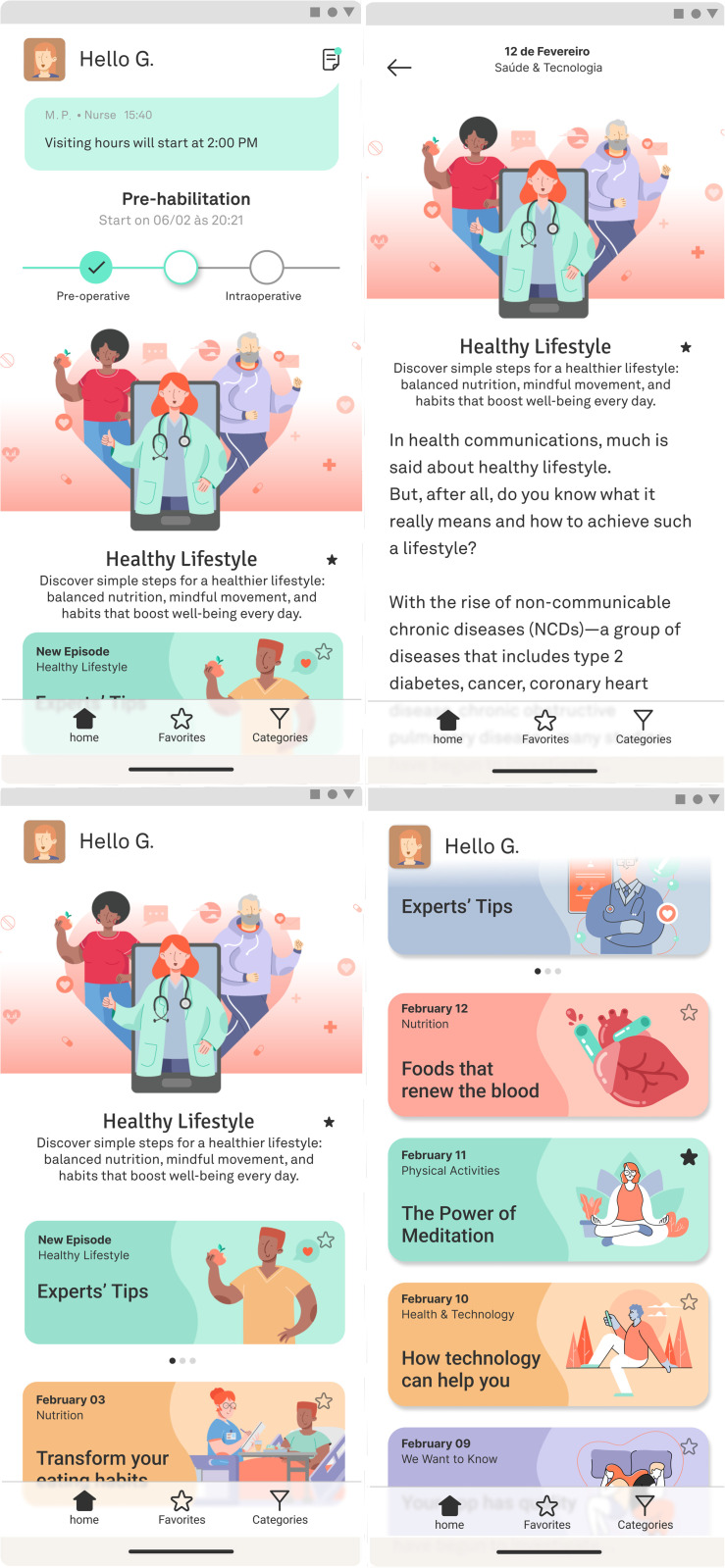
Educational module of the application for patients and families.

This module provides a user-friendly educational content in text, image and video formats, personalized for the REPLICCAR study, aiming to enhance patient and family engagement while reinforcing prehabilitation and postoperative rehabilitation guidelines (Fig 5). It includes features for scheduling messages to patients’ mobile devices at predefined dates and times, as well as tracking interaction and engagement levels. Patients can be grouped by specific parameters, such as smoking, activity level, or waiting time for surgery, enabling targeted content delivery. The module also supports surveys and facilitates communication between patients and healthcare staff. As patients progress through hospitalization stages (preoperative ward, operating room, ICU, postoperative ward, hospital discharge), their families receive real-time updates.

- Communication among the multiprofessional team

**Fig 6:**
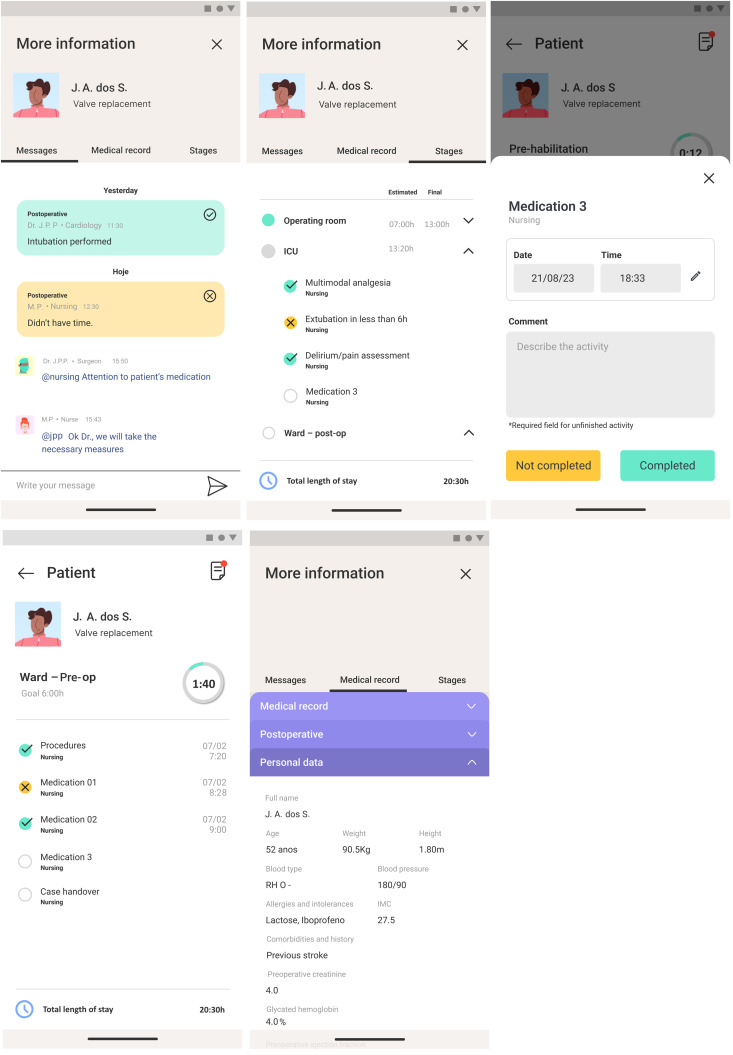
Multiprofessional care coordination team module of the application.

Facilitates coordinated communication among multidisciplinary team members regarding patients enrolled in the project pathway, including scheduling and tracking of each process step (Fig 6). This module records real-time completion of activities, monitors time spent at each stage, and ensures adherence to protocol activities.

- Activity management via Dashboard

**Fig 7:**
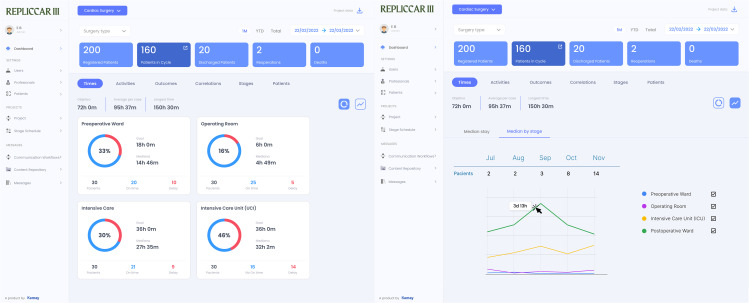
Application dashboard for monitoring activities and metrics.

An interface that enables monitoring of key metrics such as time spent per stage, adherence to activities, and patient survey results, among other indicators (Fig 7).

The patient application monitors access to educational content, while the healthcare professionals’ application tracks clinical team compliance (Fig 7).

### Study population

Inclusion criteria: patients aged ≥18 years; indication for primary isolated CABG, either elective or urgent; ownership of a personal cell phone with internet access and proficiency in its use; ability to understand and fully agree to the informed consent form.

Exclusion criteria: patients requiring concurrent surgical procedures; glycosylated hemoglobin level >8%; creatinine clearance 4%; physical or mental disabilities preventing protocol adherence; and patient or family refusal to participate.

### Ethical approval and informed consent

This study was approved by the Ethics Committee for Analysis of Research Projects (CAPPesq) at Hospital das Clínicas da Universidade de São Paulo, under approval number 77933424.8.1001.0068. Written informed consent will be obtained from all patients included in the study, regardless of the analysis period.

### Statistical analysis

Variables will be compared using the chi-square test, Student’s t-test, or Kruskal-Wallis test. Categorical variables will be presented as numbers and percentages, while continuous variables will be presented as mean ± standard deviation (SD) or median and interquartile range (IQR). All analyses will be conducted using R software version 3.1.2 [32], within an integrated development environment for statistical computing and graphics, available in precompiled binary versions for Windows. Given the stepped-wedge cluster randomized design and the potential intra-cluster correlation, mixed effect models will be applied to evaluate possible cluster and period effects.

The randomization sequence for the five clusters (participating hospitals) will be computer-generated. Allocation will be hidden at both the cluster and patient levels, with prior knowledge limited to the project leader and the team responsible for sequence generation. Blinding will be maintained for the analysis of results.

### Sample size calculation

Based on the postoperative length of stay found by Da Silva et al. [33], with a mean of 8.34 days (SD ± 10.32) and targeting a mean length of stay of 5.56 days (SD ± 10.32) after intervention, the sample calculation of REPLICCAR III was made using a two-tailed t-test for differences between two independent means. With a significance level of 5%, test power of 80%, and effect size of 0.27, the required sample size was determined to be 436 patients. Accounting for a 10% potential loss to follow-up, a final target sample size is 480 patients (240 per group). Sample size calculation was performed using G*Power software, version 3.1.9.4.

Because REPLICCAR III uses a stepped-wedge cluster randomized design, we incorporate the intra-cluster correlation (ICC), cluster size variability, and period effects (1 period = 2 months, time).

- Design and allocation

We plan a cross-sectional stepped-wedge cluster randomized design 5 clusters (hospitals, K), 6 periods that includes the control phase (T), and an average of 16 patients per cluster-period (m), totaling K × T × m = 5 × 6 × 16 = 480 patients. All models will include fixed period effects to adjust for secular trends across time.

- Correlation and cluster size variability.

Based on pilot information and published data on postoperative length-of-stay within hospitals, we assume a very low ICC = 0.001 and balanced recruitment across clusters (coefficient of variation of cluster sizes CV ≈ 0, achieved through monitoring and capped enrollment).


$$DE=\left[1+\left(m-1\right)\times ICC\right]\times \left(1+CV^{2}\right)$$

Under these assumptions, the standard design effect is:


DE≈1+15×0.001×=1.015(withCV≈0)

Therefore, with ICC = 0.001, CV ≈ 0, K = 5, T = 6, and m = 16, we will recruit N = 480 patients.

### Outcomes

The primary outcome is the difference in postoperative length of stay between the intervention and control groups. Secondary outcomes include adherence to protocol metrics during the intervention period; comparison of morbidity, mortality rates, hospital readmissions, and assessment of PROMs and PREMs between the intervention and control group at 30 days and up to 6 months after CABG.

## Discussion

CABG is a primary therapeutic approach for treating coronary artery disease and one of the most performed and studied cardiac surgeries worldwide [34]. Despite ongoing technical and scientific advancements aimed at improving outcomes [2,35,36], prolonged hospital stay after surgery remains a significant challenge for healthcare teams who seek continuous improvement in patient care. In Brazil, the average length of stay after CABG in 2021 was 11,8 days [37], incurring an estimated cost of approximately 1 billion reais [38].

Factors such as inadequate preoperative preparation, disconnected care protocols, and decision-making based on outdated concepts are related to prolonged hospital stay, negatively affecting the patient experience and increasing the risk of complications, ultimately impacting prognosis [6,39–41]. Studies indicate that extended hospitalization is associated with higher rates of postoperative morbidity and mortality, in addition to imposing a financial burden on healthcare institutions [6,7,42–44].

Despite the proven benefits of optimized recovery pathways, their implementation in cardiac surgery is still rare and inconsistent, due to barriers related to interdisciplinary team interaction and communication [45–47]. In Brazil, a pioneering group developed first series of cases based on the Enhanced Recovery After Surgery concept, in the STS EACTS LATAM conference in 2019 [48], the same year that ERAS published its first guideline for cardiac surgery (ERACS) [24]. A subsequent detailed analysis of this series reported a median postoperative length of stay of 3 days while maintaining quality and safety standards and reducing negative outcomes [1]. However, the study also highlighted challenges related to adherence to metrics and effective communication among healthcare teams.

The use of a mobile application can improve adherence to protocols by facilitating communication and monitoring of metrics, while optimizing the journey of patients undergoing CABG [13,14] and procedures in other specialties [47–49]. Successful implementation requires involvement from hospital management and comprehensive staff training. The mobile device application is presented as a tool that humanizes the use of technology, facilitating the educational content delivery to patients, enhancing communication among healthcare professionals, and providing a comprehensive view of the patient’s trajectory during hospitalization. Patient familiarity with mobile devices and internet use was considered as inclusion criteria to ensure intervention adherence. In contrast, a previous study [49] suggested that technology may not improve adherence to enhanced recovery protocols for colorectal surgery. It is important to point out that the study had varying patient assistance needs, lack of blinding, and inpatient-only data collection.

In this scenario, the Kamay application significantly supports the project, distinguishing itself from other available apps. A user-friendly design that combines text, videos and images, aiming to engage and educate patients and relatives for the perioperative phase, as well as pre- and post-rehabilitation. The professional module maps the entire perioperative cycle, categorizes patients according to their care stage, enables process checklists, alerts unmet targets and tracks key ERAS-based activity metrics, features that enhance adherence and sustainability of the care model, reducing manual workload and interpersonal strain, minimizing repeated reminders from hospital leadership. By connecting multiprofessional teams, patients and their families in real time, the platform fosters engagement that ERAS protocols alone cannot achieve. Lastly, the activity management dashboard compiles all input data into real-time graphs and reports, supporting continuous data analysis and process improvement. The modular design and dynamic updates across hospitalization stages promote transparency and shared responsibility, favoring the dissemination of an ERACS culture in daily practice.

In 2024, Dou et al. [50] published a research protocol with similarities to the proposed project; however, it did not incorporate technological resources during the intervention period. The inclusion of digital tools in our study is critical, as evidence shows that the use of applications and platforms can enhance care quality, reduce complications, and generate cost savings [14]. By integrating technology, our modernizes the approach, enabling real-time monitoring and personalized support. Technology represents the future of medicine and aligns with current trends in healthcare innovation [14].

The study will be conducted using a stepped-wedge cluster randomized design, as used by Dou et al. [50], which allows for gradual evaluation of the intervention and is particularly well-suited for service delivery interventions. This model effectively balances robust assessments with logistical limitations and reduces biases since the same clinical team treat the REPLICCAR patients during both the control and intervention phases [51]. The choice of a stepped-wedge design was also based on the well-established effectiveness of rapid recovery care pathways, which are supported by strong scientific evidence. Given this context, a traditional parallel cluster randomized trial was considered ethically inappropriate.

In view of this, our hypothesis is that the optimized recovery concept, based on the ERAS framework and combined with a real-time monitoring application, has the potential to significantly reduce hospitalization times for patients undergoing CABG surgery at major centers in the state of São Paulo, by increasing adherence to care protocol metrics. The post-pandemic context presents a favorable scenario, characterized by a repressed demand of at least 150% [52,53], which could decrease hospital exposure risk and reduce consumption of resources, while increasing bed turnover, and expanding access to healthcare within the Brazilian Unified Health System.

Given the significant challenges faced by many low- and middle-income countries (including resource limitations and patient population), the findings of this study may have relevance beyond the Brazilian context. If the hypothesis is confirmed, implementing the *Tempos Certos* enhanced recovery pathway for CABG surgery, adapted for the Brazilian context [48], associated by a digital heath tool as the Kamay app, could serve as a scalable model to improve results in diverse settings, although future research will be needed to explore its adaptation and feasibility.

After the advancements achieved through the creation and development of the REPLICCAR I and REPLICCAR II [31] databases, REPLICCAR represents an unprecedented study in CABG. This study aims to demonstrate that the use of a health application associated with an optimized recovery care protocol can reduce postoperative length of stay for patients undergoing CABG in the state of São Paulo, thereby providing greater quality and safety of treatment for individuals with cardiovascular diseases in the state.

## Supporting information

S1 File Spirit Checklist. (PDF)

S2 Table Patient-Reported Outcomes questionnaire to be collected 30 days after hospital discharge via phone call or e-mail. (DOCX)
