# Supplementary material for: The technological proposal based on enhanced recovery after cardiac surgery for changing traditional care in Latin America: REPLICCAR III Study Protocol
Source: PLoS One. 2025 Dec 31;20(12):e0338301. doi: 10.1371/journal.pone.0338301 (PMC12755796; doi:10.1371/journal.pone.0338301)
Supplement: S2 Table — (DOCX) [file pone.0338301.s002.docx]

| Type | Question | Scale |
| --- | --- | --- |
| Recovery quality | Where are you staying? | At home; At home with a nurse; At a nursing facilty |
| Anxiety | Have you been feeling anxious for the past 24 hours? | Analog scale 0-10 |
| Recovery quality | Have you experienced any new or sudden pain in any of these areas? | Chest; Shoulder; Heels; No, I do not have pain in these areas |
| Pain | While at rest, how much pain did you feel yesterday? | 0-10 point Likert scale |
| Pain | While moving, how much pain did you feel yesterday? | 0-10 point Likert scale |
| Recovery quality | Are you taking the pain medications prescribed by your doctor? | Yes; No; I forgot; No, they make me feel worse; I do not go out and I forgot to have them with me; No, I do not need them |
| Task | What was your systolic pressure yesterday? | 90 or below; 91 to 139; 140 or above |
| Task | What was your heart rate yesterday? | 69 or below; 61 to 99; 100 or above |
| Task | Did you eat healthily yesterday? | Yes; No, but I ate enough; No, I had no appetite |
| Recovery quality | Have you noticed any of these signs of dehydration? | Dark, concentrated urine; Urinating less than 4 times a day; Dry lips and mouth; Dizziness or lightheadedness; Fainting; No, I have not experienced any of these |
| Recovery quality | How were your bowel movements yesterday? | Normal; Had liquid diarrhea; Felt dry and constipated; No bowel movements yesterday; No bowel movements for 3 days or more |
| Task | Did you perform your breathing exercises yesterday? | Yes; No |
| Recovery quality | What was the highest volume you achieved in spirometry yesterday? | 500 mL or less; 1000 mL; 1500 mL; 2000 mL; 2500 mL |
| Mobility | Did you go for a walk yesterday? | Yes; No, it was raining; No, I was very tired; No, I was in too much pain |
| Recovery quality | How did you feel after your walk yesterday? | Normal; Somewhat tired; Very tired |
| Recovery quality | Did you experience any difficulty breathing while at rest? | Yes, I can barely breathe; Yes, a little; No |
| Recovery quality | Are you experiencing any difficulty urinating? | Yes, I cannot urinate; Yes, it feels like burning; No |
| Recovery quality | Have you experienced a fever of more than 37.8 °C that lasted for more than 24 hours? | Yes; No |
| Recovery quality | Have you experienced chest pain again, similar to what you felt before surgery? | Yes; No |
| Recovery quality | Have you experienced any of the following common problems after surgery? | Crackling noise in the middle of the chest; Swelling in the legs; Difficulty sleeping or nightmares; Feeling stressed or sad; I did not experienced any of these problems |
| Task | When you examine your surgical wound, do you notice any of these signs? | Increasing redness and warmth; Increasing swelling; Green or yellowish discharge from the wound; Foul odor from the wound; Bleeding that soaks the bandage; The wound is completely open; No, I am fine |
| Recovery quality | Since your last evaluation, have you needed to seek any of these medical services? | I visited my general practitioner for a problem; I had to schedule an extra appointment; I went to the emergency room; I was hospitalized; No, I did not need any services |

Adapted from Ben-Ali W et Al. [25]. Translated by REPLICCAR III research team and reviewed by a professional Portuguese-English translator.
